# Predictors and Prevalence of Severe Obstructive Sleep Apnea: A Cross-Sectional Study in Erbil, Kurdistan Region, Iraq

**DOI:** 10.7759/cureus.74055

**Published:** 2024-11-19

**Authors:** Shwan Amen, Banan Q Rasool, Aya Balisani, Dahen Tariq, Bareq S Al Lami, Maria-Alina Stefan, Shadan N Khdher, Ahmed L Mohammed, Hiba H Majeed, Paiman Taha, Dhuha A Omar, Baran K Sulaiman, Chro M Bakr

**Affiliations:** 1 Cardiology, Surgical Specialty Hospital, Erbil, IRQ; 2 Research and Development, Erbil Cardiovascular Research Center, Erbil, IRQ; 3 Internal Medicine, Erbil Teaching Hospital, Erbil, IRQ; 4 Internal Medicine, Hawler Medical University, Erbil, IRQ; 5 College of Medicine, Hawler Medical University, Erbil, IRQ; 6 General Medicine, Hawler Medical University, Erbil, IRQ; 7 Medicine, Carol Davila University of Medicine and Pharmacy, Bucharest, ROU; 8 College of Health Sciences, Hawler Medical University, Erbil, IRQ; 9 Medicine, Ministry of Health, Kurdistan Regional Government, Erbil, IRQ

**Keywords:** ahi, obstructive sleep apnea, predictors, prevalence, severe osa

## Abstract

Background

Obstructive sleep apnea (OSA) is a common sleep disorder that’s characterized by episodes of a complete or partial collapse of the upper airway with an associated decrease in oxygen saturation or arousal from sleep. According to the American Academy of Sleep Medicine (AASM), OSA is categorized based on polysomnography findings into mild, moderate, and severe.

Objectives

This study aims at determining the prevalence of the severities of OSA in Erbil, Kurdistan Region of Iraq, as well as discovering the predictors for severe OSA.

Methods

This was a cross-sectional study that was carried out from December 2021 to July 2023 on patients displaying OSA symptoms in a sleep study section of a private clinic in Erbil, Kurdistan Region of Iraq. A detailed questionnaire was designed to collect the data, and IBM SPSS Statistics for Windows, Version 26 (Released 2019; IBM Corp., Armonk, New York, United States) was used to analyze it. The polysomnography device used for the diagnosis of OSA was a Philips Respironics Alice NightOne home device, and Philips Respironics Sleepware G3 (Koninklijke Philips N.V., Amsterdam, Netherlands) was used to analyze the sleep data.

Results

A sample size of 328 OSA cases was analyzed. The results revealed a prevalence of 47% (155) for severe OSA. Apnea-hypopnea index (AHI) was negatively correlated with lowest and average oxygen saturation, while it was positively correlated with time spent with oxygen saturation under 89%, body mass index (BMI), and weight of the participants. Furthermore, stepwise multiple regression tests revealed BMI, age, gender, and heart failure as independent predictors for AHI.

Conclusion

This study highlights the links between OSA and various chronic health conditions. Furthermore, it underscores the importance of factors like age, obesity, and gender in influencing OSA severity. The identification of predictors for OSA severity can assist in risk assessment and personalized interventions.

## Introduction

Obstructive sleep apnea (OSA) is a common sleep disorder that affects an estimated 2-4% of adults worldwide. Prevalence is usually higher in certain ethnicities, such as Hispanic, Black, and Asian populations [1]. OSA is characterized by episodes of a complete (apnea) or partial collapse (hypopnea) of the upper airway with an associated decrease in oxygen saturation or arousal from sleep [2]. 

The exact cause of OSA is not fully understood. Contributing risk factors include older age, male sex, menopause, narrowed upper airway, obesity, alcohol use, smoking, nasal congestion, and genetic factors [3]. OSA is strongly associated with high body mass index (BMI); about 70% of OSA-diagnosed patients are obese, and about 40% of obese individuals are affected by OSA [4].

OSA can have a significant impact on a patient’s health and quality of life. Patients with OSA have been reported to have decreased health-related quality of life (HRQoL) in social, emotional, and physical areas [5]. The typical symptoms include morning headaches, intense daytime sleepiness, depression, fatigue, witnessed apneas and snoring, anxiety or irritation, and deficits in memory and attention with a subsequent risk of road accidents by day [6].

OSA can be diagnosed through polysomnography. The American Academy of Sleep Medicine (AASM) diagnostic criteria are 15 or more obstructive events per hour, which include apneas, hypopneas, and respiratory arousals, or five or more such events per hour along with associated symptoms such as daytime sleepiness, fatigue, and tiring sleep; and gasping, choking, loud snoring, and breathing interruptions [7].

According to AASM, OSA is categorized based on polysomnography findings into mild (five to 15 obstructive events per hour), moderate (15-30 obstructive events per hour), and severe (30 obstructive events per hour) [8]. These different categories of OSA have been found to display different clinical characteristics, with more severe OSA being associated with more comorbidities and clinical consequences [9]. In addition, the higher the severity of OSA, the lower the O_2_ saturation results are in the polysomnography results [10]. Low O_2_ saturations found in the polysomnography result have been associated with fatal clinical outcomes, such as sudden cardiac death and respiratory failure [11]. Therefore, determining the predictors for severe OSA is important in order to prevent these dire consequences.

Treatment options for OSA depend on the severity. The main treatment offered is device therapy such as continuous positive airway pressure (CPAP), which is the most effective treatment for moderate and severe OSA. CPAP delivers a stream of pressurized air through a mask that is worn over the nose and mouth while sleeping. The primary treatment for mild OSA is observation if asymptomatic. Oral appliances, positional therapy, and surgery are first-line options for symptomatic mild OSA [12].

Although there are many studies discussing the predictors and prevalence of OSA, there is a lack of such studies in the Kurdistan Region of Iraq. This study aims at determining the prevalence of the severities of OSA in Erbil, Kurdistan Region of Iraq, as well as discovering the predictors for severe OSA. 

## Materials and methods

Study design and timeline

This was a cross-sectional study that was conducted from December 2021 to July 2023.

Study setting

This study included all the patients who reported symptoms of OSA and underwent polysomnography in order to reach a diagnosis at the sleep study section in a private clinic, Dr. Shwan's Cardiology Clinic in Erbil, Kurdistan Region of Iraq. These patients included referrals from other internists as well as walk-in patients. All of the participants of the study were patients on their first visit for OSA symptoms before any treatment was carried out. The specific private clinic was chosen because it is the only sleep study center in Erbil.

Inclusion and exclusion criteria

Any patients who reported symptoms of OSA, such as morning headaches, snoring, daytime sleepiness, and fatigue, and underwent polysomnography in the clinic were included in the study. Any patients who had other possible causes of hypoxia were excluded from the study.

Sample size 

The analytical sample for the cross-sectional study consisted of 381 patients, 22 of whom were omitted due to missing polysomnography reports, evaluated with a questionnaire where all the necessary information was collected and preserved.

Questionnaire information

A structured questionnaire was used to obtain all the necessary details such as demographic information, BMI, physical activity regularity, smoking and alcohol use, presence of comorbidities (such as hypertension, diabetes mellitus, ischemic heart disease, renal failure, asthma, and thyroid diseases), presence of symptoms (including snoring, morning headache, fatigability, and/or daytime sleepiness), and polysomnography findings (apnea-hypopnea index (AHI), lowest O_2_ saturation, average O_2_ saturation, and oxygen saturation (SpO_2)_ time under 89%) (Appendix 1).

Statistical analysis

IBM SPSS Statistics for Windows, Version 26 (Released 2019; IBM Corp., Armonk, New York, United States) was used for the statistical analysis. Spearman and Pearson's correlation tests were carried out to test correlations between OSA severity and symptoms, comorbidities, and risk factors. Non-parametric tests were used to compare means between different OSA groups. Multiple stepwise linear regression tests were carried out to find predictors for OSA severity. GraphPad Prism version 9.5.0 (Insight Venture Management, LLC, New York, USA) was used to make tables and graphs. A p-value<0.05 was deemed significant.

Polysomnography

The home device used for the diagnosis of OSA was Philips Respironics Alice NightOne; the parameters that were used for the purposes of this study were AHI, lowest O_2_ saturation, average O_2_ saturation, and time spent under SpO_2_ of <89%. The program used to analyze the sleep data was Philips Respironics Sleepware G3 (Koninklijke Philips N.V., Amsterdam, Netherlands), interpreted by an experienced physician according to established guidelines. All of the studies had durations of four hours or longer; shorter studies were either repeated or discarded.

Ethical consideration

Consent was obtained from each patient directly, and their identities were kept confidential by assigning each questionnaire a numerical digit and hiding the patient’s names. This study adhered to the highest ethical standards and was approved by Dr. Shwan's Cardiology Clinic's Ethics Committee.

## Results

This was a cross-sectional retrospective study with a sample size of 359 cases. Out of them, 328 (91.4%) were diagnosed with OSA, and we ran our analysis on these 328 cases.

The majority of the participants were aged 50-70 (63.7%, n=209) followed by ages 30-49 (29.3%, n=96), >70 (5.8%, n=19), and lastly ages <30 (1.2%, n=4). Of the participants, 175 (53.4%) were female, and 153 (46.6%) were male. Around 236 (72.0%) participants were obese (BMI of 30 or more), 80 (24.4%) were overweight (BMI between 25 and 30), and 12 (3.7%) were normal weight (BMI between 18 and 25). Regarding their physical health, 251 (76.5%) participants exercised, while 77 (23.5%) participants did not. Regarding the clinical characteristics of the study population, hypertension had the highest (192, 58.5%) prevalence in those with OSA. This was followed by cardiovascular diseases, with a prevalence of 126 (38.4%) for heart failure and 113 (34.5%) for ischemic heart disease (IHD). Patients with renal failure (serum creatinine >1.5 mg/dl) had the fourth highest prevalence of disease (23.2%, n=76), followed by diabetes mellitus (22.0%, n=72). Lastly, 39 (11.9%) of the study population had thyroid problems, and 20 (6.1%) had asthma. Furthermore, 52 (15.9%) of the participants were smokers and 13 (4.0%) were alcoholics (Table 1).

**Table 1:** Baseline and clinical characteristics of the study population BMI: body mass index

Characteristics	Frequency		Percentage (%)
Age Groups			
<30	4		1.2
30-49	96		29.3
50-70	209		63.7
>70	19		5.8
Gender			
Female	175		53.4
Male	153		46.6
BMI			
Normal	12		3.7
Overweight	80		24.4
Obese	236		72.0
Exercise			
No	77		23.5
Yes	251		76.5
Hypertension			
No	136		41.5
Yes	192		58.5
Diabetes Mellitus			
No	256		78.0
Yes	72		22.0
Thyroid Diseases			
No	289		88.1
Yes	39		11.9
Asthma			
No	308		93.9
Yes	20		6.1
Ischemic Heart Disease			
No	215		65.5
Yes	113		34.5
Heart Failure			
No	202		61.6
Yes	126		38.4
Renal Failure			
No	252		76.8
Yes	76		23.2
Smoking			
No	276		84.1
Yes	52		15.9
Alcohol			
No		315	96.0
Yes		13	4.0

**Figure 1:**
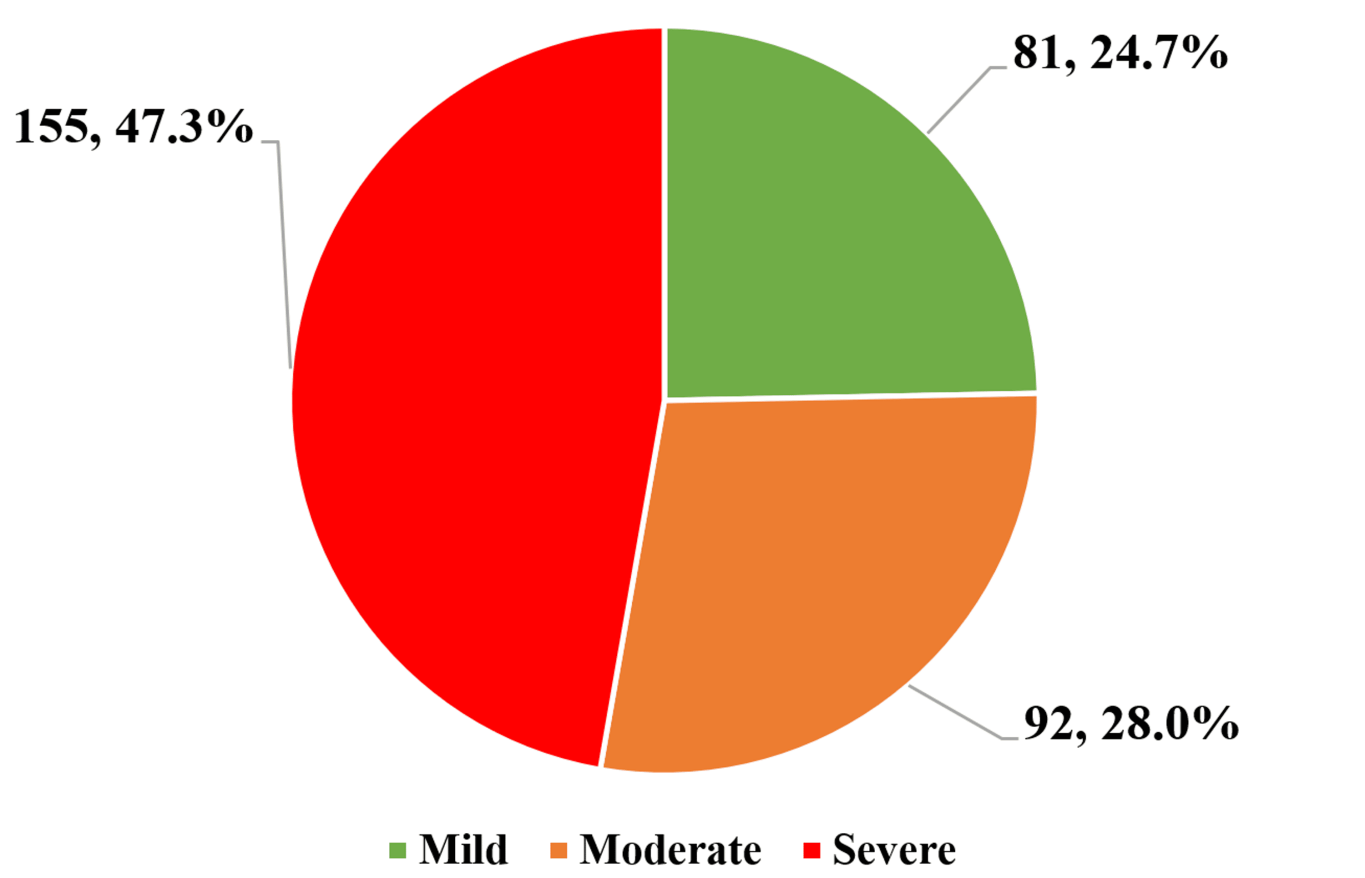
Prevalence of obstructive sleep apnea severity categories

The study population diagnosed with OSA was further categorized into mild, moderate, and severe OSA based on polysomnography findings according to AASM guidelines. The majority of the cases (155) had severe OSA (47.3%), followed by moderate OSA 92 (28.0%) and finally mild OSA 81 (24.7%) (Figure 1).

**Figure 2:**
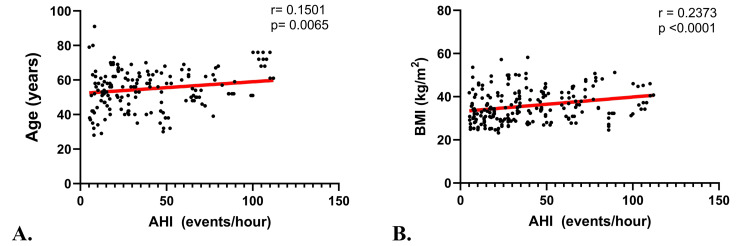
Correlation graphs between apnea-hypopnea index and (A) age and (B) body mass index

Pearson and Spearman correlation tests were carried out to test the correlation of various risk factors, clinical manifestations, and other polysomnography findings of the study population with their AHI. Results showed a moderately negative correlation that was statistically significant between AHI and lowest saturation (r=-0.63, p<0.001) as well as average saturation (r=-0.51, p<0.001). In contrast, a moderately positive correlation was found between AHI and time of SpO_2_ under 89% (the average time each patient spent with their SpO_2_<89%) with a highly significant p-value (r=0.47, p<0.001). The weight of the participants as well as their BMI had weak, positive correlations with AHI with correlation coefficients of 0.37 and 0.24, respectively; these correlations were both statistically significant (p<0.001) (Table 2, Figure 2).

**Table 2:** The correlation analysis of apnea-hypopnea index with obstructive sleep apnea risk factors and symptoms *Data was analyzed by Pearson’s correlation coefficient test; ** Data was analyzed by Spearman’s correlation coefficient test; BMI: body mass index

Parameters	Apnea-Hypopnea Index	
	OSA patients (n=328)	
	r	p-value
Lowest saturation(%)*	-0.63	<0.001
Average saturation(%)*	-0.51	<0.001
Time SpO_2 _under 89(%)*	0.47	<0.001
Age(years)*	0.15	0.006
Weight(kg)*	0.37	<0.001
BMI*	0.24	<0.001
Gender	0.17	0.002
Morning headache**	0.19	0.001
Heart failure **	-0.11	0.045
Hypertension**	0.1	0.073
Exercise**	-0.03	0.550
Snoring **	0.06	0.262
Daytime sleepiness**	0.07	0.218

**Figure 3:**
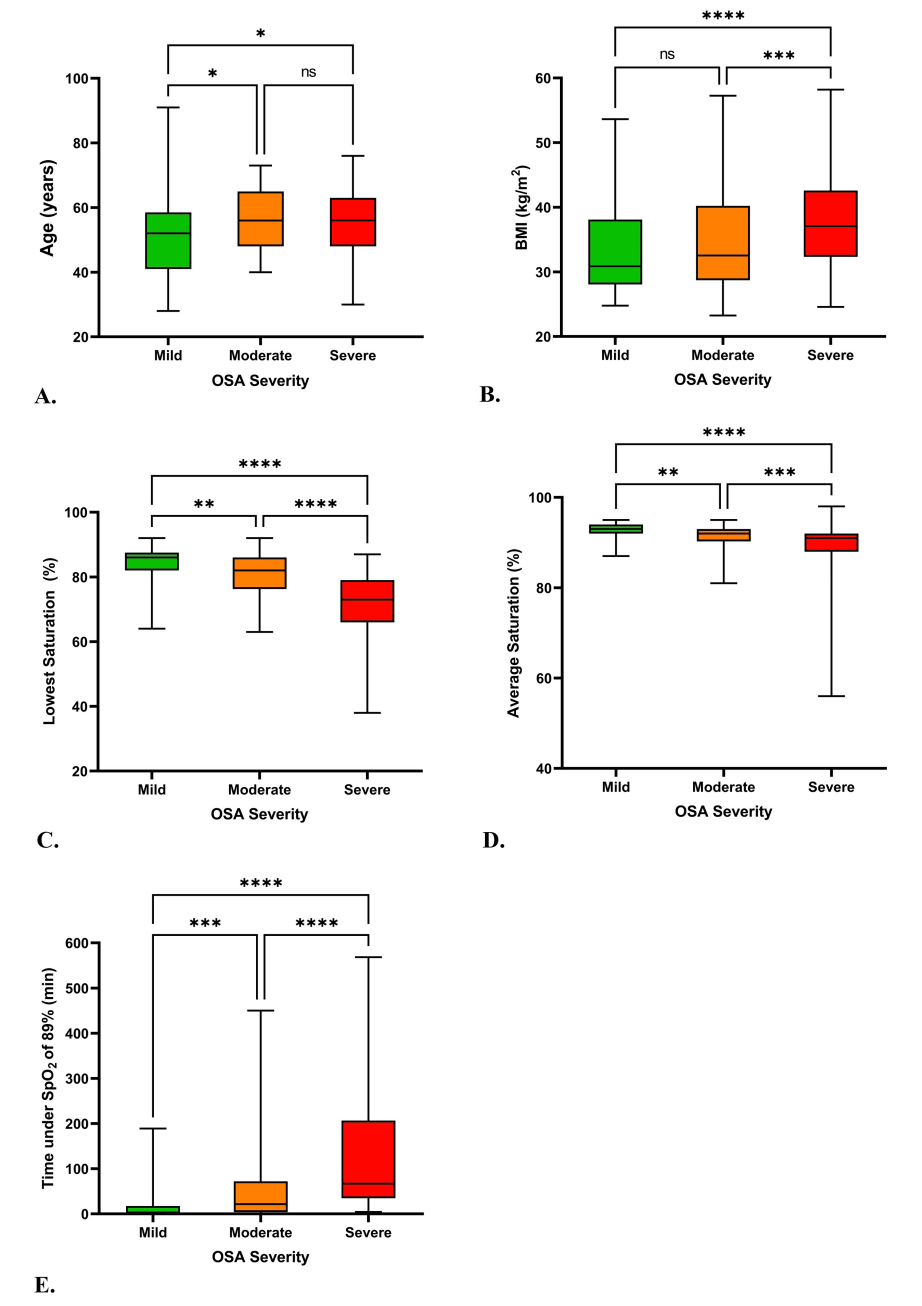
Differences in age (A), BMI (B), lowest saturation (C), average saturation (D), and time spent under 89% saturation (E) between the OSA severity groups ns P > 0.05, * P ≤ 0.05, ** P ≤ 0.01, *** P ≤ 0.001, **** P ≤ 0.0001; BMI: body mass index

A Kruskal-Wallis ANOVA test was performed to compare the means of various variables among different OSA groups. The results revealed significant differences in the means of age (p=0.0212), BMI (p<0.0001), lowest saturation (p<0.0001), average saturation (p<0.0001), and time spent under 89% saturation (p<0.0001). The exact differences are shown in Figure 3.

Stepwise multiple regression was performed to determine the predictors of AHI. The results showed BMI, gender, age, and heart failure as independent predictors of AHI. These were significant with p-values<0.001 for BMI, gender, and heart failure and a p-value of 0.001 for age (Table 3).

**Table 3:** Stepwise multiple regression analysis reveals BMI, gender, age, and heart failure as predictors for apnea-hypopnea index (AHI) in the study population The excluded variables are ischemic heart disease (IHD), renal failure (RF), asthma, thyroid disease, exercise, alcohol consumption, hypertension, and diabetes mellitus. (B: The unstandardized coefficient regression, beta: the standardized coefficient regression, R2: the coefficient of determination (squared correlation)).

Model	B	Beta	R^2^	Adjusted R^2^	P-value
1			0.056	0.053	<0.001
Constant	6.104				
BMI	0.835	0.237			
2			0.130	0.125	<0.001
Constant	-30.392				
BMI	1.231	0.350			
Gender	15.283	0.294			
3			0.161	0.153	0.001
Constant	-53.719				
BMI	1.214	0.345			
Gender	16.924	0.325			
Age	0.394	0.178			
4			0.213	0.204	<0.001
Constant	-52.687				
BMI	1.329	0.378			
Gender	18.354	0.353			
Age	0.599	0.270			
Heart Failure	-13.285	-0.249			

## Discussion

The findings of this study provide a comprehensive understanding of OSA within the Erbil Region of Kurdistan, Iraq. By examining the prevalence, clinical characteristics, correlations between OSA severity and risk factors, and predictors of OSA severity, this study contributes valuable insights into the epidemiology and clinical implications of OSA in this population.

Prevalence and clinical characteristics

The high prevalence of OSA within the study population, with over 90% of participants diagnosed with the disorder, underscores its significance as a widespread health concern in the Erbil region. This prevalence aligns with global trends, reaffirming the substantial burden OSA poses to public health. Furthermore, the varying distribution across age groups highlights the association between advancing age and OSA, which can be attributed to the fact that older individuals possess certain anatomical changes and diminished muscle tone in the upper airway.

The clinical characteristics of the OSA-diagnosed population offer valuable insights into the profile of individuals affected by the disorder. The presence of comorbid conditions such as hypertension, heart failure, renal failure, and diabetes mellitus among a significant proportion of participants shows a linking tool that ties OSA to cardiovascular and metabolic disorders. These findings highlight the importance of addressing OSA within the broader context of patient's health and emphasize the need for integrated management strategies.

Certain comorbid conditions were found to be interlinked with the prevalence of OSA. For example, obesity was seen playing a major role in the development of OSA, considering that up to 60% of obese patients suffer from OSA [13]. Within the selected population sample of our study, 236 people (72.0%) were obese, 80 (24.4%) people were overweight, and 12 (3.7%) were normal weight. Nearly all of the cases with an increased BMI had a major increase in apnea/hypopnea events/hour (known as events/hour), and patients with a BMI of more than 30 experience an increased severity of OSA.

Hypertension is a prevalent characteristic in OSA patients. Different subtypes of hypertension, such as essential or resistant hypertension, are seen to have a role when discussing OSA severity. It is estimated that 38% (essential) to 71% (resistant) of hypertensive patients possess comorbid OSA [14]. On the other hand, around 50% of OSA patients are hypertensive [15]. Similarly, our study showed that hypertension had the highest (58.5%) prevalence among participants with OSA. Induced arousal from sleep, strong negative intrathoracic pressure swings, hypercapnia, and upper airway obstruction all cause rapid increases in peripheral vasoconstriction, subsequently raising the blood pressure. Even though hypertension and OSA are common diseases with multiple etiological factors and coexist in cardiovascular comorbidities (e.g., obesity and metabolic syndrome), an independent relationship between these two seems to exist [14]. OSA can lead to metabolic dysfunction through several pathways, including intermittent hypoxia, sleep fragmentation, sympathetic activation, and inflammation. Intermittent hypoxia, which is a hallmark of OSA, can cause oxidative stress and inflammation, leading to endothelial dysfunction and insulin resistance. Sleep fragmentation, another common feature of OSA, can disrupt the normal circadian rhythm and lead to dysregulation of glucose metabolism and appetite hormones. Sympathetic activation, which is also associated with OSA, can increase blood pressure and heart rate, leading to hypertension and cardiovascular disease. Inflammation, which is a common feature of metabolic syndrome, can be exacerbated by OSA, leading to a vicious cycle of metabolic dysfunction and cardiovascular disease.

Similarly, the prevalence of OSA among patients with type 2 diabetes mellitus (T2DM) ranges from 55% to 86% [16]. About 22.0% of our patients with OSA had T2DM and patients with T2DM are at a higher risk of developing OSA when compared to patients without diabetes [17,18]. The cardinal features of OSA, including intermittent hypoxemia and sleep fragmentation, have been linked to abnormal glucose metabolism in laboratory-based experiments. OSA has also been linked to the development of incident T2DM, and the relationship between OSA and T2DM may be bidirectional in nature given that diabetic neuropathy can affect central control of respiration and upper airway neural reflexes, promoting sleep-disordered breathing [16].

Ample evidence has demonstrated an elevated risk of coronary artery disease (CAD) in patients with OSA in spite of other co-existing cardiovascular disease (CVD) comorbidities [19]. Among our study population, 38.4% of patients with OSA were positive for heart failure, which in turn meant their ejection fraction was below the 40% threshold and 34.5% for IHD. In particular, the prevalence of sleep-disordered breathing in CAD patients is doubled compared with the general population, and more than 70% of patients with acute coronary heart disease are afflicted with undiagnosed OSA [20]. Furthermore, OSA has been associated with a higher risk of nocturnal ischemic events and appears to trigger the incidence of sudden death at nighttime. It has been reported that 32% of OSA patients would suffer from a myocardial infarction (MI) attack between 12 AM and 6 PM, compared with 7% of non-OSA patients [21].

Correlation between OSA severity and the conventional determinants

The results of the correlation analyses between OSA severity, as indicated by the AHI, and various risk factors provide crucial insights into the relationship between OSA and its determinants. The negative correlations between AHI and lowest and average oxygen saturation levels highlight the strong association between OSA severity and nocturnal hypoxemia. This observation is consistent with the understanding that OSA-related airway obstructions lead to intermittent hypoxemia during sleep. Similar findings are found, such as the one conducted by Kong et al. [11], where they also found that the lowest SpO_2_ was negatively correlated with the severity of OSA.

The positive correlation between AHI and time spent during the at-home sleep studies with oxygen saturation below 89% further underscores the clinical significance of prolonged oxygen desaturation with how severe OSA can be. Again, the same findings were found in similar studies, such as the one conducted by Kong et al. [11] and the Medical Advisory Secretariat [22]. This finding carries implications for individuals experiencing these prolonged periods of hypoxemia, as it may contribute to a range of adverse health outcomes, including cardiovascular complications.

The observed weak positive correlations between AHI and weight as well as BMI align with well-established links between obesity and OSA. The study by Jordan et al. [23] has shown that a reduction of up to 10 kilograms in body mass can produce a reduction in AHI of roughly five events/hour. This can be attributed to the fact that excess adiposity contributes to the narrowing of the upper airway and thus increases the mechanical load on respiratory muscles, predisposing individuals to airway collapse during sleep.

Variances among different risk factors and their significance in relation to OSA severity

The Kruskal-Wallis ANOVA test, a non-parametric alternative to traditional ANOVA, was adeptly employed to assess the significance of differences in various variables across the distinct OSA severity groups. This analysis revealed noteworthy patterns that contribute significantly to our understanding of OSA progression and its clinical implications.

Among the standout findings was the age distribution among the OSA severity groups. The observed statistically significant difference (p=0.021) in age across the severity categories signifies an intrinsic relationship between age and OSA severity. This outcome aligns with existing research that indicates a higher prevalence and severity of OSA among older individuals [24,25]. It suggests that advancing age may act as a precipitating factor for the exacerbation of OSA symptoms, possibly due to the cumulative effects of anatomical changes and other risk factors. Decreased muscle tone, increased fat deposition, and decreased lung function are some of the anatomical changes seen. These changes can lead to a narrowing of the upper airway and an increased risk of airway collapse during sleep, which can contribute to the development of OSA.

Additionally, the test illuminated the role of BMI in influencing OSA severity. The highly significant differences in BMI across OSA severity groups (p<0.005) highlight the well-established link between obesity and OSA [26]. This finding accentuates the importance of weight management strategies as a potential avenue for alleviating OSA severity and improving overall patient outcomes.

Furthermore, the Kruskal-Wallis ANOVA test unveiled significant differences in key polysomnography parameters: lowest saturation, average saturation, and time spent under 89% saturation (P< 0.005). These results emphasize the clinical relevance of these parameters in characterizing OSA severity. Notably, the time spent under 89% saturation exhibited a significant positive correlation with OSA severity. This correlation highlights the potential clinical implication of prolonged oxygen desaturation periods, indicative of severe OSA cases. Such findings were also seen in a study conducted by Wali et al. [27]. Such correlations will help physicians when it comes to patient management, making polysomnography one of the cornerstones of patient care in suspected cases of OSA [28].

Predictors of OSA severity

Our analysis revealed several significant predictors of OSA severity, including BMI, gender, age, and cardiovascular diseases. These predictors provide valuable insights into the multifactorial nature of OSA development and severity. BMI, a well-established risk factor for OSA, showed a positive correlation with OSA severity, consistent with the findings from studies conducted previously that emphasized the relationship between obesity and the likelihood of developing OSA [23,26]. Gender emerged as another predictor, with females potentially exhibiting different OSA risk profiles compared to males. This finding resonates with studies indicating variations in OSA prevalence and characteristics between genders [29,30].

Age also played a role in predicting OSA severity, suggesting that advancing age may contribute to a higher risk of severe OSA. This aligns with the well-documented age-related changes in upper airway anatomy and muscle tone that could facilitate airway collapse during sleep [24,25]. The significant association between heart failure and OSA severity highlights the intricate interplay between cardiovascular health and sleep-disordered breathing, a relationship that has been increasingly recognized in the literature [31]. During apneic events, there is a decrease in oxygen saturation and an increase in carbon dioxide levels, leading to a cascade of physiological responses, including sympathetic activation, increased heart rate, and increased blood pressure. These responses can cause an increase in afterload on the heart and a decrease in cardiac output, leading to impaired cardiac function and heart failure. In addition, the repeated and acute swings in intrathoracic pressure induced by apneic events during sleep can cause an increase in venous return and left ventricular afterload, accompanied by stroke volume decrease, further contributing to heart failure. Furthermore, activation of the sympathetic nervous system secondary to hypoxia and arousal leads to tachycardia, peripheral vasoconstriction, and higher myocardial oxygen consumption, which can also contribute to heart failure [31]. This finding highlights the importance of comprehensive assessments and targeted interventions for individuals with both heart failure and OSA to mitigate potential adverse outcomes.

Clinical implications and recommendations

The implications of the study's findings extend to clinical practice and public health strategies. The identified predictors for AHI-BMI, gender, age, and heart failure hold the potential to guide risk assessment and inform tailored interventions. Incorporating these predictors into clinical assessments could help identify individuals at higher risk for severe OSA, facilitating early diagnosis and targeted management approaches. Public health measures aimed at reducing the incidence of raised BMI and heart failure among the population can help reduce the rate of severe OSA and its subsequent consequences.

The associations between OSA and comorbid conditions, such as cardiovascular diseases and status, emphasize the importance of a comprehensive approach to patient care. Healthcare providers should consider the presence of these conditions when evaluating individuals with suspected OSA, as diagnosing and treating OSA earlier alongside associated comorbidities could lead to improved overall outcomes in the event efficient follow-ups were followed.

Limitations and future directions

Several limitations of the study warrant consideration. The cross-sectional design restricts the ability to infer causality, highlighting the need for longitudinal investigations to explore temporal relationships between risk factors and OSA development. Reliance on self-reported data introduces the possibility of recall bias, underscoring the value of validating self-reported variables through objective measures. The single-center nature of the study and the exclusion of specific patient groups could limit the generalizability of findings to broader populations.

Future research endeavors could encompass longitudinal studies that establish causal relationships and investigate the impact of OSA treatment on associated conditions. Multicenter studies involving diverse populations would enhance the external validity of findings. Employing objective measures for variables such as smoking, alcohol use, and physical activity could help mitigate potential biases.

## Conclusions

This study conducted in Erbil, Kurdistan Region of Iraq, provides valuable insights into OSA within this population. It highlights the need for a more comprehensive patient care approach, recognizing the links between OSA and various chronic health conditions. Furthermore, it underscores the importance of factors like age, obesity, and gender in influencing OSA severity. The identification of predictors for OSA severity can assist in risk assessment and personalized interventions. Overall, this research provides valuable insights into OSA’s impact on public health in this region and emphasizes the importance of comprehensive patient management.

## Appendices

Appendix 1

i. Demographics:

Age: __________                                   Gender: __________

Weight (kg): ________                           Height (Meters): __________

Occupation: _________                          Residency (Province): ________

Do you exercise? Yes     ​No

iia. Past medical history:

Comorbidities: 

Hypertension

Diabetes mellitus

Ischemic heart disease

Thyroid disease

Asthma

Heart failure

Renal failure

Alcohol:  Yes No

Smoking: Yes No

iib. Symptoms:

Snoring

Morning headache 

Fatigability

Daytime sleepiness

iii. Polysomnography findings:

• AHI: ___________

• Lowest O_2_ saturation: _________

• Average saturation: __________

• Time spent under SpO_2_ of < 89%: _______
